# Enhancing pollination is more effective than increased conventional agriculture inputs for improving watermelon yields

**DOI:** 10.1002/ece3.6278

**Published:** 2020-04-23

**Authors:** Thomas Sawe, Katrine Eldegard, Ørjan Totland, Samora Macrice, Anders Nielsen

**Affiliations:** ^1^ Faculty of Environmental Sciences and Natural Resource Management Norwegian University of Life Sciences Ås Norway; ^2^ Department of Biological Sciences University of Bergen Bergen Norway; ^3^ Department of Ecosystems and Conservation Sokoine University of Agriculture Morogoro Tanzania; ^4^ Department of Landscape and Biodiversity Norwegian Institute of Bioeconomy Research (NIBIO) Ås Norway; ^5^ Department of Biosciences Centre for Ecological and Evolutionary Synthesis (CEES) University of Oslo Oslo Norway

**Keywords:** agriculture, brix, fertilizer, fruit‐quality, irrigation, pollinator‐limitation

## Abstract

Agricultural practices to improve yields in small‐scale farms in Africa usually focus on improving growing conditions for the crops by applying fertilizers, irrigation, and/or pesticides. This may, however, have limited effect on yield if the availability of effective pollinators is too low. In this study, we established an experiment to test whether soil fertility, soil moisture, and/or pollination was limiting watermelon (*Citrullus lanatus*) yields in Northern Tanzania. We subjected the experimental field to common farming practices while we treated selected plants with extrafertilizer applications, increased irrigation and/or extra pollination in a three‐way factorial experiment. One week before harvest, we assessed yield from each plant, quantified as the number of mature fruits and their weights. We also assessed fruit shape since this may affect the market price. For the first fruit ripening on each plant, we also assessed sugar content (brix) and flesh color as measures of fruit quality for human consumption. Extra pollination significantly increased the probability of a plant producing a second fruit of a size the farmer could sell at the market, and also the fruit sugar content, whereas additional fertilizer applications or increased irrigation did not improve yields. In addition, we did not find significant effects of increased fertilizer or watering on fruit sugar, weight, or color. We concluded that, insufficient pollination is limiting watermelon yields in our experiment and we suggest that this may be a common situation in sub‐Saharan Africa. It is therefore critically important that small‐scale farmers understand the role of pollinators and understand their importance for agricultural production. Agricultural policies to improve yields in developing countries should therefore also include measures to improve pollination services by giving education and advisory services to farmers on how to develop pollinator‐friendly habitats in agricultural landscapes.

## Graphical abstract

Extra pollination increased the probability of a watermelon plant producing a second sellable fruit as well as fruit sugar content. Moreover, we learnt that, increased number of pollinators does not necessarily increase fruit set. Finally, we learnt that, increased fertilizer and watering/irrigation did not influence yield quality or quantity.

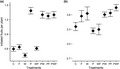

## 1 INTRODUCTION

The role played by animal pollinators in agricultural production is largely unknown by the majority of local farmers in sub‐Saharan Africa (Eardley, Roth, Clarke, Buchmann, & Gemmill, 2006; Gollin, 2014), while at the same time it attracts enormous attention in the northern hemisphere (Timberlake & Morgan, 2018). Governments and agricultural stakeholders in sub‐Saharan Africa have emphasized the significance of improving soil conditions through fertilization and artificial irrigation to maximize yields in this region (Gollin, 2014; Güneralp, Lwasa, Masundire, Parnell, & Seto, 2017; Lema, Machunda, & Njau, 2014), whereas the potential contribution of pollination for optimizing crop yield has been largely overlooked. According to Klein et al. (2007), 35% of global food production comes from animal pollinated crops, and the Intergovernmental Science‐Policy Platform on Biodiversity and Ecosystem Services (IPBES) have estimated the direct economic contribution of animal pollinators to global agricultural production to be in the range of 5%–8% (IPBES, 2016). This might seem low, but it constitutes a crucial part of the human diet, because most animal pollinated food plants—such as vegetables and fruits—have high nutritional value, whereas cereals, such as wheat, rice, and maize, are wind or self‐pollinated (Sulewska et al., 2014). Moreover, insect‐pollinated crops have higher economic value and might thus contribute more to farmers' income and countries' gross domestic product (Gallai, Salles, Settele, & Vaissière, 2009). Insect pollination can also significantly improve fruit quality such as fruit shape, sugar content, and shelf life (Klatt et al., 2014).

According to Hopwood et al. (2016), most research on crop pollination over the last 20 years have been conducted in developed countries and have mainly focused on how insect pollination alone can improve yield. There is a general lack of studies addressing multiple yield‐limiting factors, such as insufficient pollination, fertilization, and/or water availability. Consequently, the degree to which pollination regulates yield in cropping systems is still debated (Ghazoul, 2007; Kremen, Daily, Klein, & Scofield, 2008), and the role of pollination relative to water and nutrient limitation, seed quality, pests, and diseases is poorly understood (Klein, Hendrix, Clough, Scofield, & Kremen, 2015), especially in tropical agro‐ecosystems.

In sub‐Saharan Africa, most researchers emphasize low levels of soil nutrients and insufficient rainfall as the main factors responsible for low agriculture production (Sheahan & Barrett, 2017). For these reasons, improving soil conditions and investing in irrigation schemes are the focuses for agricultural development to improve yields. This focus on fertilizers and water to increase yield in staple crops such as rice, wheat, maize, and potatoes has underpinned the lack of research on pollination deficits in insect‐pollinated cash crops. In Tanzania, for example, the use of fertilizer has increased from an average of 5.5 kg/ha in 2004/2005 to 9 kg/ha in 2009/2010 (Mather, Waized, Ndyetabula, Temu, & Minde, 2016). This is, however, far below levels reported in Southern Asia (129.4 kg/ha), South East Asia (109.6 kg/ha), and Latin America (104.8 kg/ha; Senkoro et al., 2017). Efforts to improve irrigation schemes have also been implemented in Tanzania. According to Mdee, Harrison, Mdee, Mdee, and Bahati (2014), the agricultural area under irrigation in Tanzania has expanded from 150,000 ha in 2003 to 460,000 ha in 2013 and is expected to reach 1 million ha in 2020.

In addition to abiotic factors, insufficient animal‐pollination can also put limitations on yields in animal‐pollinated crops, since pollen availability can affect fruit and seed set (Delaplane, Mayer, & Mayer, 2000; Willmer, 2011) and fruit quality (Gajc‐Wolska, Kowalczyk, Mikas, & Drajski, 2011; Klatt et al., 2014). However, in most cases, the effects of biotic and abiotic agricultural inputs have been studied independently. Manipulating fertilization and water availability in combination with pollination experiments is rarely done (but see Klein et al., 2015; Marini et al., 2015), although this is crucial for understanding the potential of these factors, separately, or in combination, for improving yields.

It is common practice among watermelon growers in North East Tanzania to fertilize their plants at least once during the growing season and irrigate at least once per week (Sawe et al., personal communication). This is in addition to other common farm practices such as pesticide spraying and weeding. Generally, different type varieties of pesticides are used at different rates, depending on type of pests, affordability, and knowledge (Sawe et al., personal communication). Watermelon (*Citrullus lanatus* Thunb., Cucurbitaceae) is self‐compatible and monoecious, and thus highly dependent on insect pollination for optimal yield (Bomfim, Bezerra, Nunes, Freitas, & Aragão, 2015; Brewer, 1974; Sanford & Ellis, 2016). Watermelon has become a vital cash crop in sub‐Saharan Africa as its market value has recently increased due to growing demands (van Ittersum et al., 2016; Makuya, Mpenda, & Ndyetabula, 2017), providing households with an extrasource of income (Makuya et al., 2017). The main watermelon cultivars (Sukari F1 hybrid and Pato F1) used in the area has the potential of producing two (3–5 kg) fruits per plant. We did, however, observe that most of the second ripening fruits were too small to achieve a good market price (<1.5 kg), and none of the plants produced more than two fruits reaching this size (Sawe, Nielsen, & Eldegard, 2020; Sawe, Nielsen, Totland, Macrice, & Eldegard, 2020). Most of the local watermelon growers suggest that low levels of fertilizer and irrigation limit their yields (Sawe, Nielsen, & Eldegard, 2020; Sawe, Nielsen, Totland, et al., 2020).

In this study, we aimed to assess the relative contribution of enhancing pollination to watermelon yield,—compared with increasing fertilization and irrigation beyond current levels of agricultural inputs by local farmers. We established an experiment and tested the effects of the following three treatments; (a) extra pollination, (b) extra fertilization, and (c) extra watering, as well as all possible treatment combinations. We compared the plants subjected to the treatments to control plants receiving standard agricultural practice and natural levels of pollination from the local pollinator community. We hypothesized that the combination of extra pollination, extrairrigation, and extra fertilization would have positive effects on the quantity and quality of watermelon yields. We tested the effects of our three main treatments—and all possible interactions—on fruit initiation, fruit weight, fruit set, fruit sugar content, fruit shape, and fruit flesh color.

## 2 MATERIALS AND METHODS

### 2.1 Study area

In August 2017, we established an experiment at Mererani in the Simanjiro‐Manyara region in Northern Tanzania (3°36′9.98″ S, 36°54′37.83″ E). We selected this particular area because it houses many watermelon growers with well‐established irrigation systems. Maize is, however, the main agriculture crop in this area. Vegetables and fruits are usually grown after the maize harvest or in relatively small agricultural gardens. The area is semi‐arid with a mean annual temperature of 24.7°C and an annual rainfall of 906 mm (Tanzania Meteorological Agency, 2016). The landscape is generally flat and dominated by naturally occurring *Acacia* trees in nonagriculture and residential areas.

### 2.2 Experimental design

**FIGURE 1:**
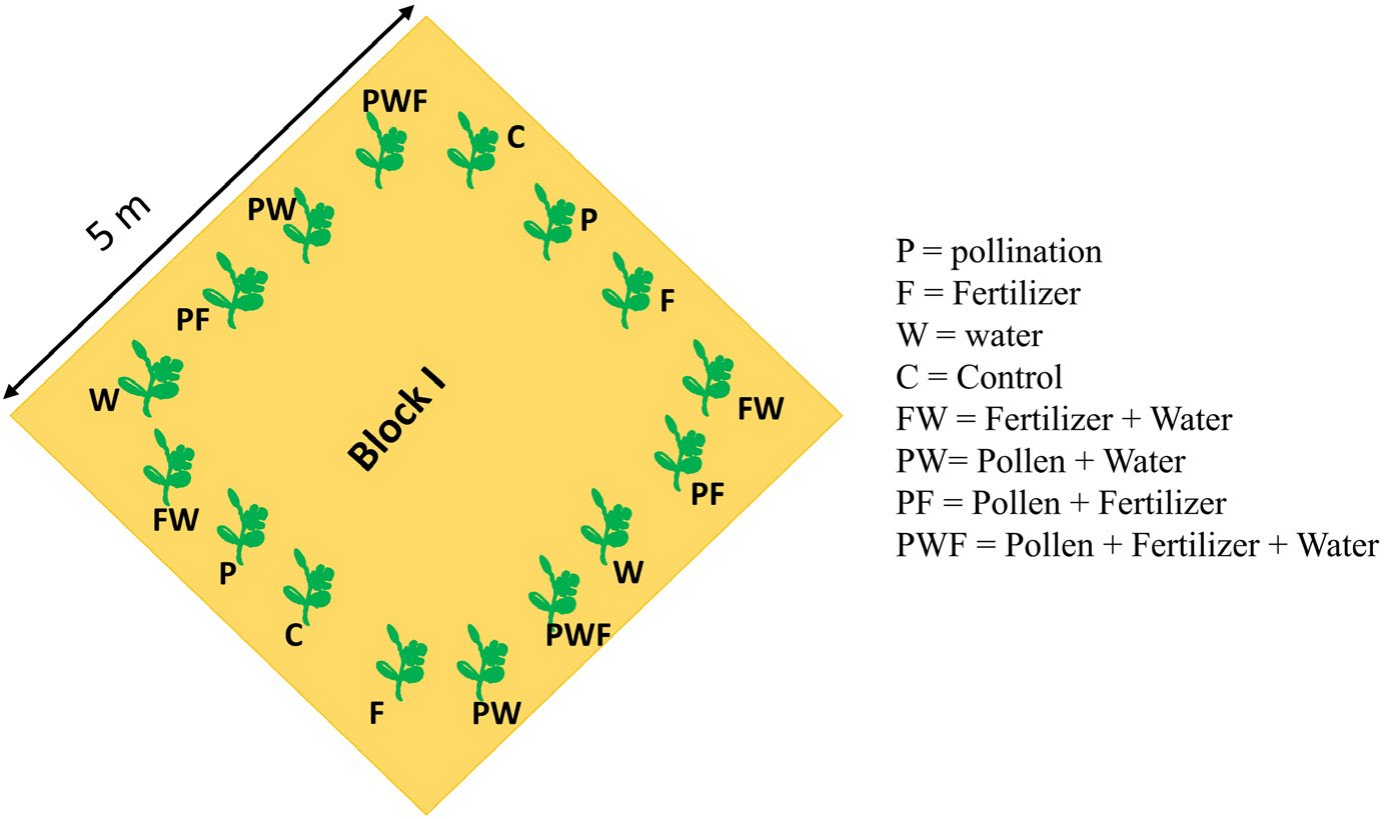
Illustration of the experiment layout with all the treatment combinations, which were the same for all the 21 replicate blocks

To test the effect of the experimental treatments (extra pollination, extra fertilization, and extra watering) and treatment interactions on watermelon yields, we prepared a garden of about 0.2 ha by dividing it into 21 square blocks of 25 m^2^ each. In each block, we planted 16 seeds of watermelon (F1 Sukari hybrid) at a distance of 1 m from each other (Figure 1) as proposed by seed manufacture and as a practice commonly adopted by most local farmers. Upon germination, we randomly selected two plants as control; similarly, we randomly assigned each treatment and all possible treatment combinations to two plants in each block.

#### 2.2.1 Control

We adopted the practice used by the majority of the watermelon growers around our study area as the control to mimic the regular local farming practices. This includes two rounds of fertilizer applications (10 g/plant of UREA‐YaraMila after germination; and 20 g/plant Nitrogen, Phosphorus, and Potassium [NPK‐YaraMila] during blossom), spraying of pesticides, irrigation, and weeding. It is common for farmers to use different types of pesticides for the same or different pest and at different stage of crop development. We subjected all plants in our experiment to the common local field practice. In this experiment, we used the following pesticides Abamectin, Megasin, Imadacloprid (Imida C), Cypercal 50 EC, Xantho, and Atakan C. On average, all plants were sprayed 2 and 4 times per week before and after flowering, respectively. The two control plants received no further treatment, whereas each of the other plants selected for the experiment received one of the—or a combination of the—following treatments;

#### 2.2.2 Extra pollination

From the onset of flowering, we conducted daily observations and registered each time a new female flower emerged on the plants. As soon as the flower opened, we hand pollinated it by rubbing it with anthers loaded with pollen. We collected anthers from male flowers from the same and different plants, ensuring a mixed pollen load that more realistically mimic the natural pollination conditions in this plant. We extrapollinated all emerging female flowers for 3 weeks, resulting in a minimum of three and maximum of six female flowers being extrapollinated per plant.

#### 2.2.3 Extra watering

After onset of germination, each plant subjected to the extra watering treatment received an extraliter of water between the two weekly regular irrigating events.

#### 2.2.4 Extra fertilization

For all plants subjected to the extra fertilization treatment, we added 10 g of Urea 1 week after the first regular application of fertilizer, and 20 g of NPK 1 week after the second regular application. This extra fertilization corresponds to a doubling of the regular amount of fertilizers applied by local farmers. Fertilizer addition was preceded by watering to dissolve the fertilizer. Urea and NPK are the major type of fertilizer used by local farmers, moreover, soils in this region are known for Nitrogen and Phosphorous deficiency (Okalebo et al., 2007).

### 2.3 Data collection

#### 2.3.1 Fruit initiation set and yield quantification

To assess the effect of our treatments on fruit initiation, we counted initiated fruits on all plants twice. We conducted the first assessment toward the end of the second week of flowering and the second at the end of the fourth week of flowering.

One week before harvesting fruits, we counted the number of mature fruits per plant. We also measured the weight of each fruit—using an electronic balance (model: Gourd shaped portable electronic scale, precision = 0.0005 kg)—and categorized them as the first, second, or third, based on the order of appearance of the flower they developed from. We only recorded fruits with weights above 1.5 kg because the farmers consider smaller fruits as unsellable at the market.

#### 2.3.2 Fruit quality assessment

We assessed sugar content of aqueous solution (brix), flesh color, and fruit shape as indicators of fruit quality. We assessed the fruit flesh color and brix from 48 fruits by randomly selecting six fruits from each treatment combination and control (we only selected among the first fruits appearing since not all plants produced more than one fruit). We juiced each fruit and determined brix using a refractometer (model: Grinding Mix Cutting Fluids) at Nelson Mandela Institute of Science and Technology in Arusha, Tanzania. Before juicing the fruit, we bisected the fruit and categorized the color of the flesh as either, “deep red” (high quality), “red” or “pale yellow” (low quality). In addition, we categorized the shape of each fruit as either “normal shape,” “mild misshaped,” or “misshaped”, since fruit shape affects the market price.

### 2.4 Data analysis

We conducted initial exploratory analyses of the dataset following (Zuur, Ieno, & Elphick, 2010) to check for outliers and to explore relationships between response variables (i.e., number, weights, fruit shape, and sugar content of fruits) and the explanatory variable treatment. We used the statistical software R version 3.3.3 for windows (R Core Team, 2017) for all statistical analyses. To build generalized linear mixed models (GLMMs), we used the lme4 library version 1.1‐19 (Bates, Mächler, Bolker, & Walker, 2015). We used GLMMs with Poisson error distribution and log link function to assess the effect of our treatments on the number of initiated fruits. We included block as a random factor in the models to account for any among‐blocks variability.

We analyzed how fruit weight and brix varied in response to our treatments using separate linear mixed models (LMMs), including block as a random factor in the models. Fruit order (first or second fruit) was included as a fixed effect covariate. Using the multinomial function in the nnet library (Venables, & Ripley, 2002) and function in the car library (Fox & Weisberg, 2011), we also analyzed likelihood of (type of) treatment predicting fruit shape and fruit color (both being categorical response variables with three levels).

Since our plants had at least one marketable fruit and since most of the subsequent ripening fruits did not attain market quality, we estimated the probability of our plants producing a second fruit that could be sold at the market. We assigned plants with two sellable fruits as “success” and plants with only one or no fruits as “failure.” We used a GLMM with binomial error distribution and log link function to fit a model including treatment as explanatory variable and included block as a random factor.

Using ANOVA function from car library, we run ANOVA of the developed models to test the significance difference between treatments and control as well the significance of the interaction effects. We report Wald chi‐squared tests, associated degrees of freedom and test statistics, in which the estimated effect of each main treatment and all treatment interactions, are compared with the control reference level. Treatment levels with associated *p*‐values <.05 were assessed as statistically different from the control treatment.

## 3 RESULTS

### 3.1 Fruit initiation

**FIGURE 2:**
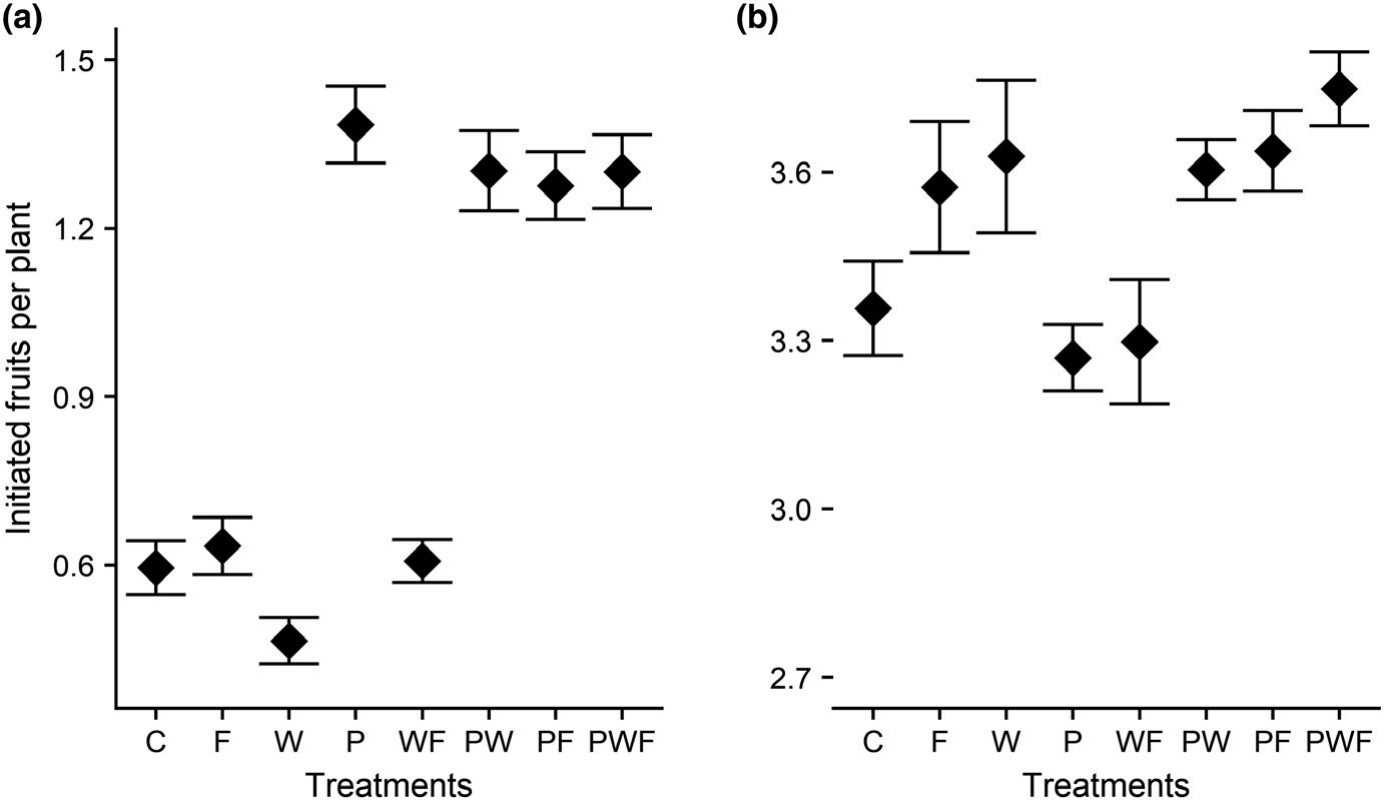
Number of initiated fruits per plant in the (a) 2nd week and the (b) 4th week after blossom. C, control; F, extra fertilizer; P, extra pollination; W, extra water. Other letter combinations correspond to different combined treatments of the three basic treatments. Points and associated error bars are observed means and standard errors

Average number of initiated fruits 2 weeks after blossom—across all the treatments—was 0.5 (*SE* ± 0.9; Figure 2a). Extra pollination significantly increased the probability of initiating fruit (Table 1); average number of initiated fruits in plants receiving extrapollinated treatment was more than twice as high as for plants receiving only natural pollination (Figure 2a). In contrast, neither extrafertilizer nor extrawater significantly affected initial fruit set 2 weeks after onset of flowering. A similar analysis of number of initiated fruits 4 weeks after onset of flowering revealed that the average number of initiated fruits was higher than at the first fruit set assessment (2 weeks after onset of flowering) across all treatments 3.4 (*SE* ± 0.4; Figure 2b). However, none of the treatments had a significant influence on fruit initiation at this stage (Table 1).

**TABLE 1:** Main and interaction effects of the applied treatments on number of initiated fruits per plant during first and second initial fruits assessment in a watermelon field in Northern Tanzania. ANOVA output of a full generalized linear mixed model with Poisson error distribution and log link function. Response variable was number of initiated fruits. F, extrafertilizer; P, extra pollination; W, extrawater

Explanatory variables	χ^2^	*df*	*P* (>χ^2^)
First fruit set assessment (2nd week postblossom)			
Intercept (control)	0.14	1	.04
F	0.11	1	.74
P	43.24	1	<.01
W	0.02	1	.89
F × P	3.36	1	.07
F × W	0.41	1	.52
P × W	0.11	1	.77
F × P × W	0.43	1	.51
Second fruit set assessment (4th week postblossom)			
Intercept (control)	1,163.06	1	<.01
F	0.82	1	.37
P	5.96	1	.01
W	0.29	1	.59
F × P	3.46	1	.06
F × W	1.17	1	.27
P × W	2.03	1	.15
F × P × W	0.71	1	.40

### 3.2 Fruit weight

**FIGURE 3:**
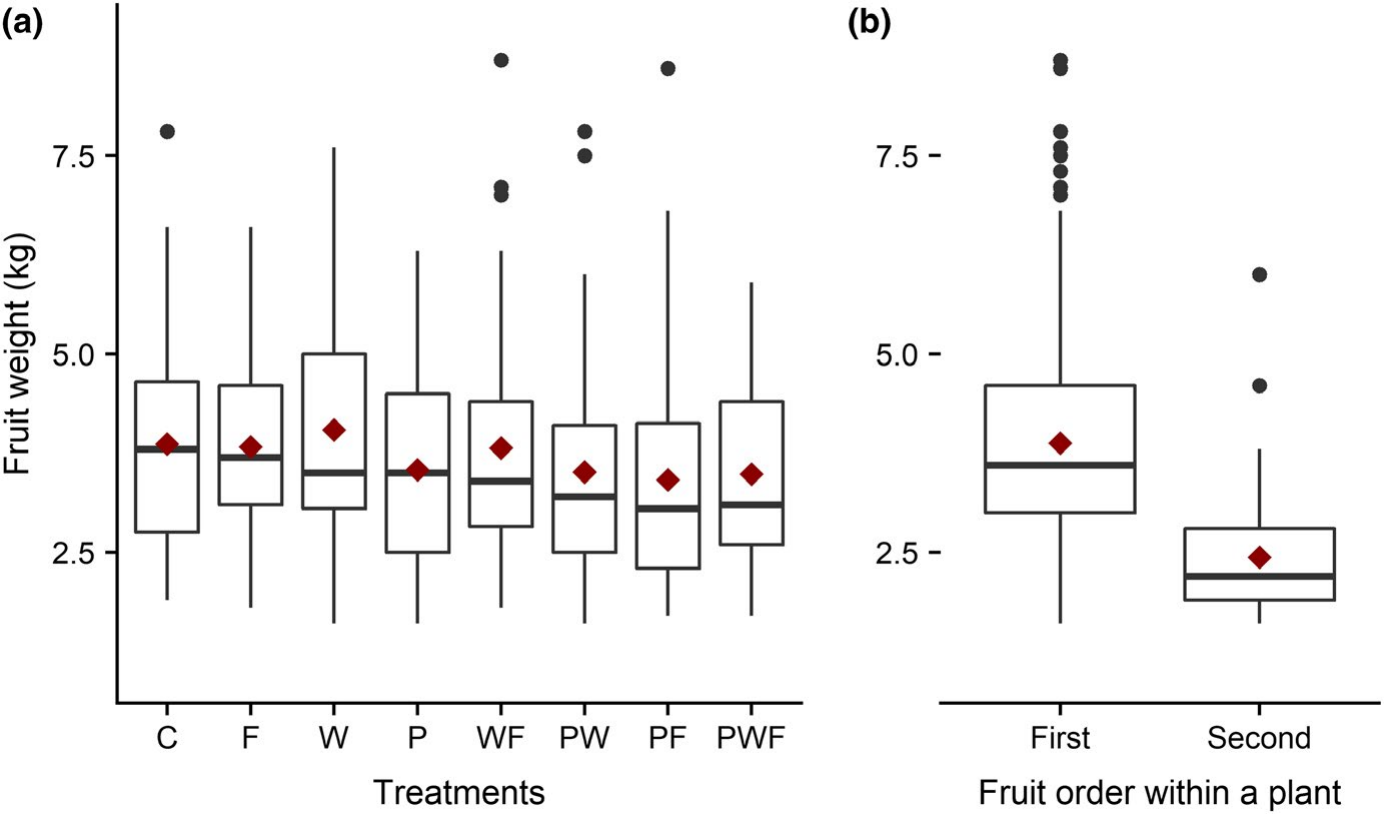
Fruit weight (a) in different treatments and (b) of first and second fruit on the same watermelon plant. C, control; F, extra fertilizer; P, extra pollination; W, extra water. Other letter combinations correspond to different combined treatments of the three basic treatments. Boxplots showing observed medians (midline), observed means (red diamonds), and the 75th and 25th percentiles (upper and lower limits of the box). The whiskers extend *up to* 1.5 times the interquartile range from the top (bottom) of the box to the furthest weight within that distance; if there are any data beyond that distance, they are represented individually as points

Average fruit weight across all treatment was 3.7 kg (Figure 3a). Neither of the treatments affected fruit weight, but the second fruit was 42% lighter than the first (Table 2; Figure 3b).

**TABLE 2:** Main and interaction effects of the applied treatments and fruit order (first or second fruit) on fruit weight in a watermelon field in Northern Tanzania. ANOVA output of a mixed model with Gaussian error distribution and identity link function. Response variable was fruit weight (kg). F, extrafertilizer; P, extra pollination; W, extrawater

	χ^2^	*df*	*P* (>χ^2^)
Intercept	383.15	1	<.01
P	0.16	1	.69
F	0.03	1	.86
W	0.22	1	.64
P × F	0.24	1	.62
P × W	0.00	1	.95
F × W	0.16	1	.69
P × W × F	0.43	1	.51
Fruit no	54.58	1	<.01

### 3.3 Probability of producing a second marketable fruit

**FIGURE 4:**
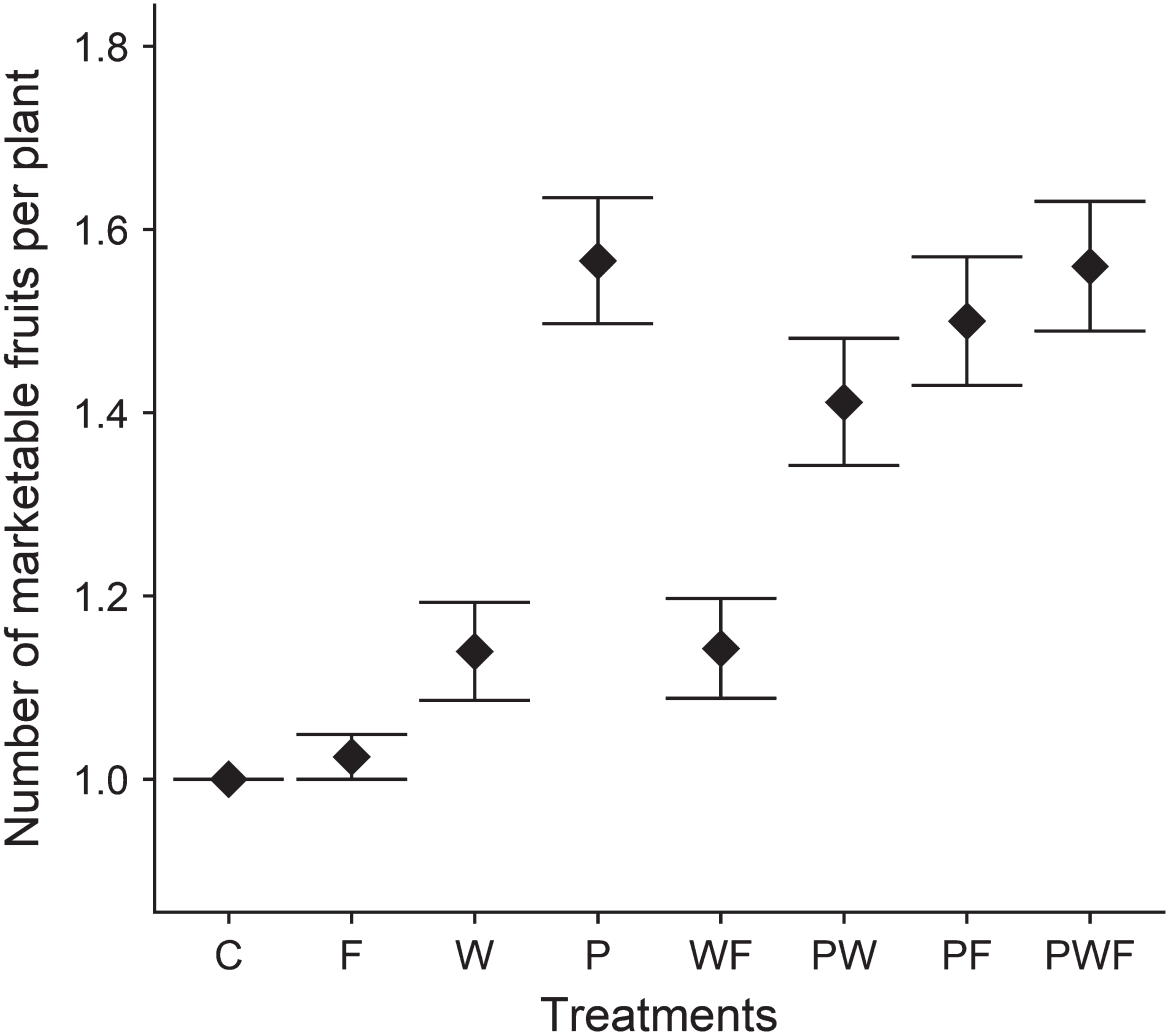
Average number of marketable watermelon fruits per plant. C, control; F, extra fertilizer; P, extra pollination; W, extra water. Other letter combinations correspond to different combined treatments of the three basic treatments. Points and associated error bars are observed means and standard errors

Irrespective of treatment, all plants produced at least one marketable fruit. In our analyses, we therefore focused on the probability of producing a second marketable fruit, as none of the plants had more than two marketable fruits at the time of harvest. Overall, 43% of the plants produced two fruits. The average number of marketable fruits per plant across the treatments was 1.1 (*SE* ± 0.6); but there was a substantial difference among treatments, whereby plants receiving extra pollination treatment had 20% higher probability of producing a second marketable fruit (Table 3). In contrast, we observed no significant effects of either extrawater or extrafertilizer (Figure 4).

**TABLE 3:** Main and interaction effects of applied treatments on the probability of a plant individual developing a second marketable fruit in a watermelon field in Northern Tanzania. ANOVA output of a full generalized linear mixed model with binomial error distribution and log it link function, with binary response (fruit vs. no fruit). F, extrafertilizer; P, extra pollination; W, extrawater

	χ^2^	*df*	*P* (>χ^2^)
Intercept	41.34	1	<.01
F	0.67	1	.41
P	4.67	1	.03
W	0.16	1	.69
F × P	0.37	1	.54
F × W	0.64	1	.42
P × W	0.00	1	.99
F × P × W	0.40	1	.52

### 3.4 Fruit quality

**FIGURE 5:**
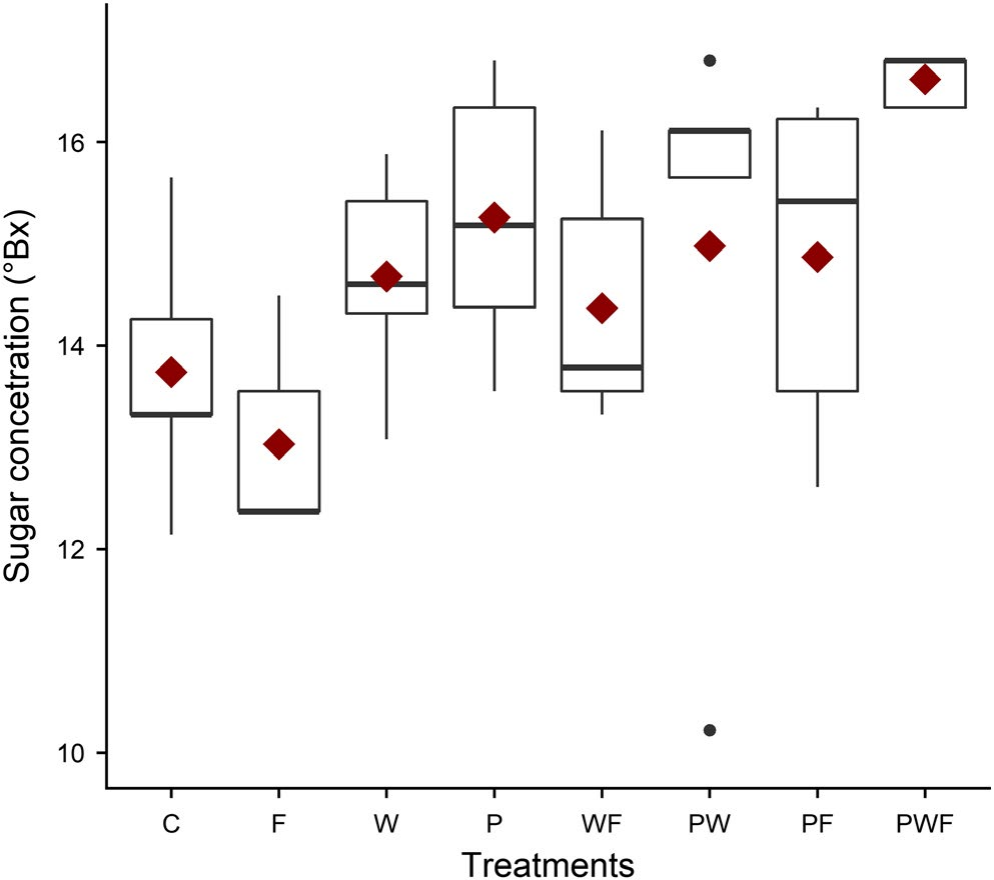
Sugar concentration in fruits. C, control; F, extra fertilizer; P, extra pollination; W, extra water. Other letter combinations correspond to different combined treatments of the three basic treatments. Boxplots showing observed medians (midline) and observed means (red diamonds)

Average amount of fruit sugar content was 13.6°Bx (*SE* ± 0.68; Figure 5), extra pollination treatment significantly increased the sugar content by approximately 10%, compared to the control treatment, while neither extrawater, extrafertilizer, nor their interaction had any effect (Table 4).

**TABLE 4:** Main and interaction effects of the applied treatments on the amount of sugar (brix) in the fruits in a watermelon field in Northern Tanzania. ANOVA output of a linear mixed model with Gaussian error distribution and identity link function. Response variable was sugar content (brix). F, extrafertilizer; P, extra pollination; W, extrawater

	χ^2^	*df*	*P* (>χ^2^)
Intercept	473.78	1	<.01
F	1.53	1	.21
P	4.46	1	.03
W	2.33	1	.12
F × P	1.42	1	.23
F × W	1.58	1	.20
P × W	0.00	1	.97
F × P × W	0.94	1	.33

## 4 DISCUSSION

Our results suggest that under current fertilizer application and irrigation schemes, insufficient pollination is limiting watermelon yield, in particular the probability of a plant producing a second sellable fruit. In contrast, we found that increased fertilization and irrigation levels, that is, increased beyond the levels applied by local farmers, did not improve watermelon yields in our experimental garden in northern Tanzania, neither in terms of quantity nor quality.

Most farmers in sub‐Saharan Africa are small holders with limited access to fertilizers and irrigation due to limited monetary and technological resources. Indeed, when asked about the causes of decline in agriculture production (not specified to type of crop), soil nutrients were the most frequently mentioned factor by local farmers in our study area (60% of 147 interviewed farmers (Sawe, Nielsen, & Eldegard, 2020; Sawe, Nielsen, Totland, et al., 2020). No one of the interviewed farmers mentioned insufficient pollination as a potential cause (Sawe Nielsen, & Eldegard, 2020a; Sawe, Nielsen, Totland, et al., 2020).

We observed higher initial fruit set in extrapollinated plants at the first assessment, and this indicates insufficient pollination early in the flowering season. This might be a result of a low flower density that fails to attract sufficient pollinators during the early stages of flowering (Essenberg, 2012). Experimentally increased nutrient and water availability did not affect fruit initiation, suggesting that current levels of watering and fertilization are sufficient for initial fruit set. During the second fruit initiation assessment, we observed a general increase in fruit set, and at this stage extra pollination did not enhance fruit initiation. This indicates that natural levels of insect pollination increased later in the flowering season. We suggest that increased flower density within the watermelon field later in the flowering season attracted more flower visitors to the field from surrounding areas (Hegland, 2014; Nielsen et al., 2012; Russo, DeBarros, Yang, Shea, & Mortensen, 2013). We also observed a negative effect of the combined treatment of water and fertilizer addition on fruit initiation during the second assessment. In a previous study, we found separate negative relationships between—respectively—increased soil moisture and soil potassium concentrations and the probability of watermelon plants initiating fruits (Sawe, Nielsen, Totland, et al., 2020). Findings from the current experiment suggest that the water and fertilizer levels normally applied by farmers (i.e., those received by the control plants) are above the plant threshold requirements for fruit initiation.

Neither extra pollination, fertilization nor watering affected fruit weight in our experiment. This does not necessarily indicate that fertilizer, water, and pollination are unimportant for watermelon fruit development. However, our results suggest that current levels of fertilizer addition, irrigation, and insect pollination are not limiting fruit weight. This contradicts the findings of Sabo, Wailare, Aliyu, Jari, and Shuaibu. (2013), who observed that fertilizer addition caused heavier watermelons in Nigeria, and those of Erdem and Yuksel (2003) and Fuentes et al. (2018) who found that fruit weight of watermelon increased with irrigation. Brewer (1974) found an increase in fruit weight in watermelon in response to increased flower visitation rates, suggesting that pollination might play a role also for fruit size. Studies on other crops, such as tomatoes, kiwi, apples, and strawberries, have also found that fruit weight increases with enhanced pollination (Abrol, Gorka, Ansari, Al‐Ghamdi, & Al‐Kahtani, 2017; Çolak, Şahinler, & İslamoğlu, 2017; Miñarro & Twizell, 2015).

The first fruits to emerge were heavier than the second fruit at the time of harvest, irrespective of treatment. Most of the second fruits were not mature, and they might have grown to a larger size if given more time to develop. However, in our study area, farmers harvest the watermelon fields only once, due to the limited yield after the main harvest and the high labor costs related to harvesting.

Extra pollination significantly increased the probability of a plant producing a second marketable fruit, while additional water or fertilizer had no effect. Several other studies have shown that fruit set increases with insect pollination in other insect‐pollinated crops (Garibaldi et al., 2013; Klein, Steffan‐Dewenter, & Tscharntke, 2003; Klein et al., 2007). The role of pollination on physiological mechanisms driving resource allocation during fruit development within a plant is well understood (Klatt et al., 2014; Roussos, Denaxa, & Damvakaris, 2009; Wietzke et al., 2018). Watermelon plants can inhibit the development of additional flowers and fruits and allocate their resources to the first initiated fruits (Delaplane et al., 2000; Mussen & Thorp, 1997; Sanford & Ellis, 2016). This suggests that increasing pollinator availability may not necessarily increase fruit initiation and development since plants allocate their resource to early initiated fruits. Therefore, since farmers harvest only once, early fruit initiation is crucial since fruits initiating later will not reach marketable size by the time of harvest. In our first assessment of fruit initiation, we found increased fruit set in extrapollinated plants, whereas in the second assessment, fruit initiation did not differ among treatments. This suggests that the probability of developing a second marketable fruit is constrained by pollinator availability early in the flowering season. We suggest that in our study system, this is not related to the effective pollination period. We suggest that such early‐season pollen limitation may be due to density‐dependent processes affecting our focal plants' attractiveness as a forage resource for the local pollinator community. Presence of other plants such as *Acacia* trees and other flowering agricultural crops around our study site could be an explanation for limited pollinator visitation in watermelon flowers. This hypothesized relationship between pollinator attractiveness at particular times during flowering, and number of fruits produced, might also be relevant for other insect‐pollinated crops, but we are not aware of other studies addressing this issue.

Previous studies of pollination of watermelon plants have proposed deploying honeybee hives on commencement and throughout the blossom period to increase the chance of all flowers being pollinated (Taha & Bayoumi, 2009). In addition, Adlerz (1966) suggested that, increasing number of honeybees increase resource competition and hence time spent per flower. Sawe et al. (in preparation) found that unmanaged honeybees were the main (87%) visitors of watermelon flowers in this region; on average 0.46 (± 0.02 *SE*) flower visits by honeybees per 10 min were observed (*N* = 23 gardens). This means that, watermelon growers can benefit both from honey and improved pollination of their crops by hanging honeybee hives around their watermelon fields.

Extra pollination increased fruit sugar concentration, while increased watering and fertilizer application did not. This is in line with other studies documenting positive effects of pollination on fruit sugar content in oilseed rape (Bommarco, Marini, & Vaissière, 2012), cucumber (Gajc‐Wolska et al., 2011), strawberries (Klatt et al., 2014), and muskmelon (Al‐Mefleh, Samarah, Zaitoun, & Al‐Ghzawi, 2012). In contrast, Cabello, Castellanos, Romojaro, Martinez‐Madrid, and Ribas (2009) suggested moderate use of water and nitrogen fertilizer on watermelon since they did not find any positive effects on fruit quality, including sugar concentration. On the other hand, for tomatoes, no (Arbex de Castro Vilas Boas et al., 2017) and even negative effects (Delazari et al., 2016) of increased watering on sugar concentration have been reported. These contrasting results imply that optimal watering and fertilization regimes for improving fruit sugar content in watermelon and other fruits depend on local environmental conditions.

None of our treatments affected fruit flesh color or fruit shape. Both color and shape are important qualities that influence the market price of watermelons and other fruits. Positive effects of pollination services on fruit shape have been reported in, for example, apples and raspberries (Çolak et al., 2017; Garratt et al., 2014; Matsumoto, Soejima, & Maejima, 2012; Pashte & Kulkarni, 2015; Sáez, Morales, Ramos, & Aizen, 2014). Sufficient pollination can therefore increase farmer's revenue through increased fruit quality. We found misshaped fruits on some of our experimental plants, but since the treatments did not affect the probability of misshape, we suggest that other factors, such as frugivorous insects, might play a more important role for fruit shape than pollination, soil nutrients, and water availability.

## 5 CONCLUSION

We have shown that increase in conventional agricultural inputs (increased fertilization and water) beyond the levels typically applied by local farmers had no effect on the numbers weight or quality of watermelon fruits produced in Northern Tanzania. In contrast, enhanced pollination early in the flowering season increased the number of fruits that attained market size, and fruits from extrapollinated flowers had higher sugar content. Thus, insufficient insect pollination is probably the main limiting factor for optimal yield in our study area. Our results suggest that there is a substantial need of a higher awareness of insect pollination as a crucial factor to increase agricultural production in Northern Tanzania, both among local farmers and agricultural authorities, and most likely also in other parts of sub‐Saharan Africa. We therefore suggest that agricultural authorities encourage a mind shift among local farmers from focusing mainly on nutrients and water addition to considering insect pollination as an important factor for improving yield. Moreover, agriculture authorities should help local farmers to develop management strategies, which will enhance pollinator availability from the early flowering stage. This can be achieved through; improvement of local pollinator habitats (Aslan, Liang, Galindo, Kimberly, & Topete, 2016), deployment of honeybees hives (Hoover & Ovinge, 2018), and increasing flowering resources such as flower strips to attract pollinators (Rundlöf, Lundin, & Bommarco, 2018) and at the same time ensuring low or no competition for flower resources (Holzschuh, Dormann, Tscharntke, & Steffan‐Dewenter, 2011).

## CONFLICT OF INTEREST

None.

## AUTHOR CONTRIBUTION

**Thomas Corodius Sawe:** Data curation (lead); formal analysis (lead); investigation (lead); methodology (supporting); writing – original draft (lead); writing – review and editing (lead). **Katrine Eldegard:** Funding acquisition (lead); project administration (lead); resources (lead); supervision (equal); writing – review and editing (supporting). **Ørjan Totland:** Conceptualization (lead); methodology (supporting); supervision (supporting); writing – review and editing (supporting). **Samora Macrice:** Supervision (supporting); writing – review and editing (supporting). **Anders Nielsen:** Conceptualization (lead); formal analysis (supporting); methodology (lead); supervision (supporting); writing – review and editing (supporting).

## Data Availability

Data are available from the Dryad Digital Repository (https://doi.org/10.5061/dryad.2fqz612m2).
